# Supplementary figures and images for: An assistive computer vision tool to automatically detect changes in fish behavior in response to ambient odor
Source: Sci Rep. 2021 Jan 13;11:1002. doi: 10.1038/s41598-020-79772-3 (PMC7806584; doi:10.1038/s41598-020-79772-3)

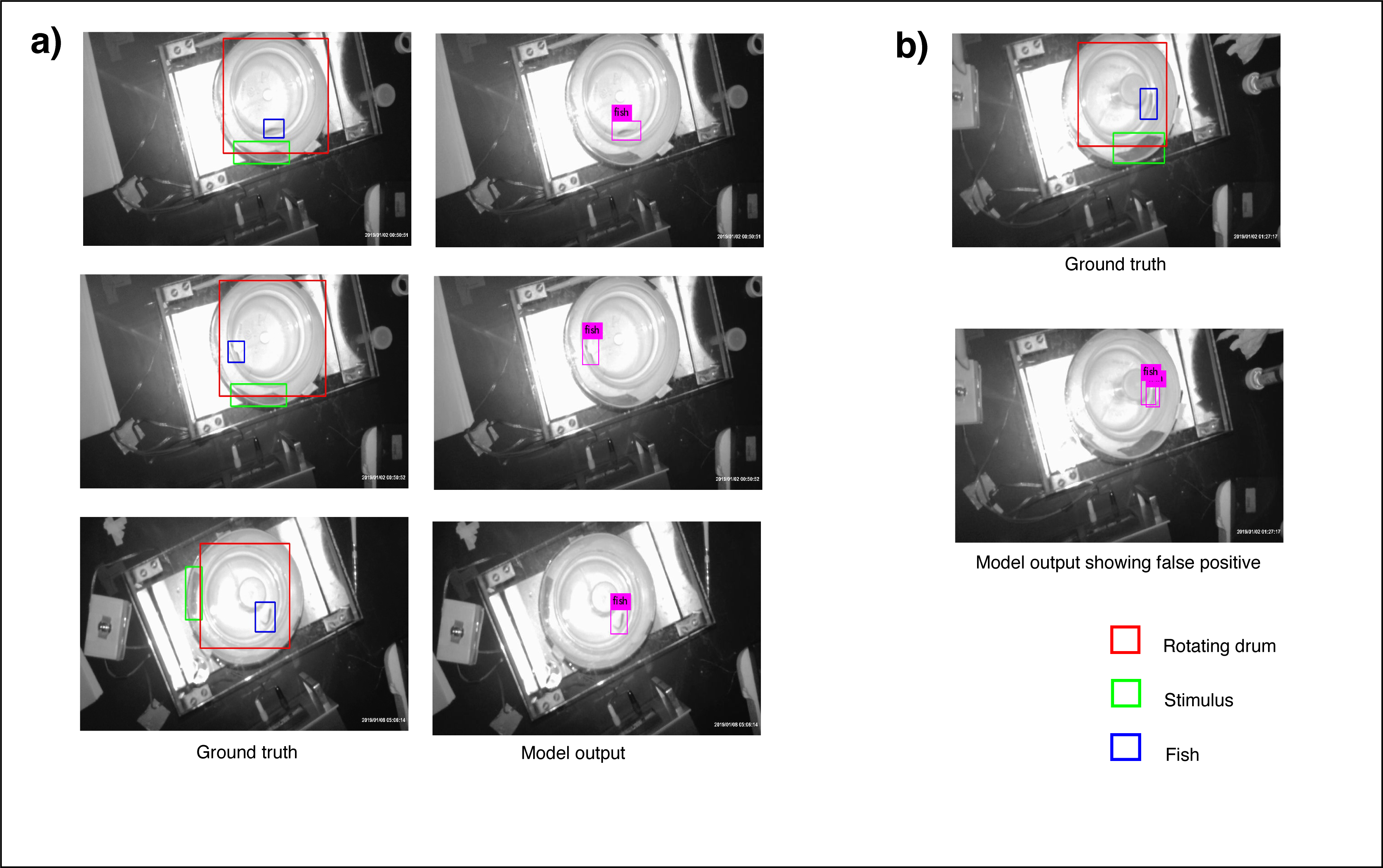

Supplement: Supplementary file 1 — Supplementary Figure 1. [file 41598_2020_79772_MOESM1_ESM.png]

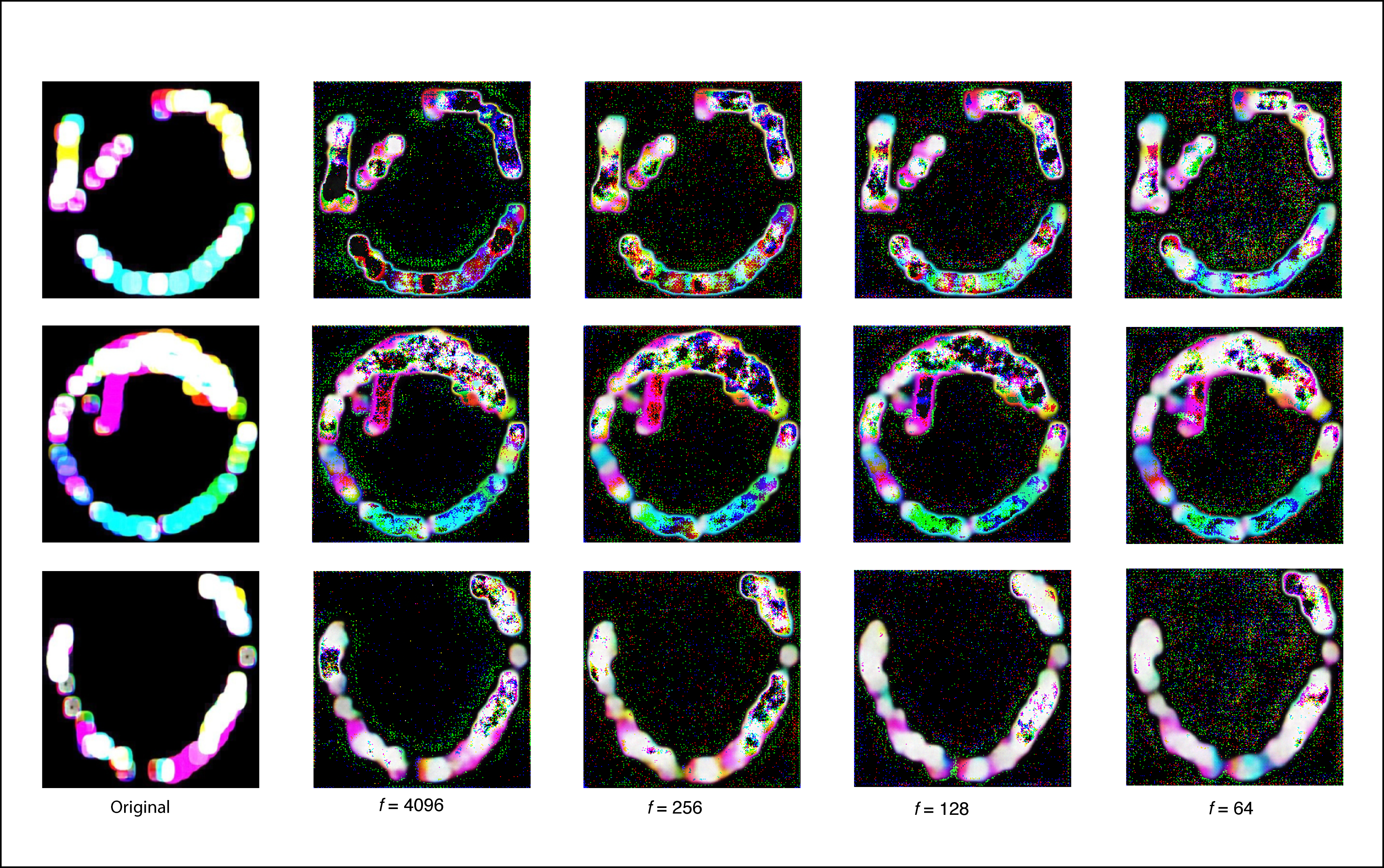

Supplement: Supplementary file 2 — Supplementary Figure 2. [file 41598_2020_79772_MOESM2_ESM.png]

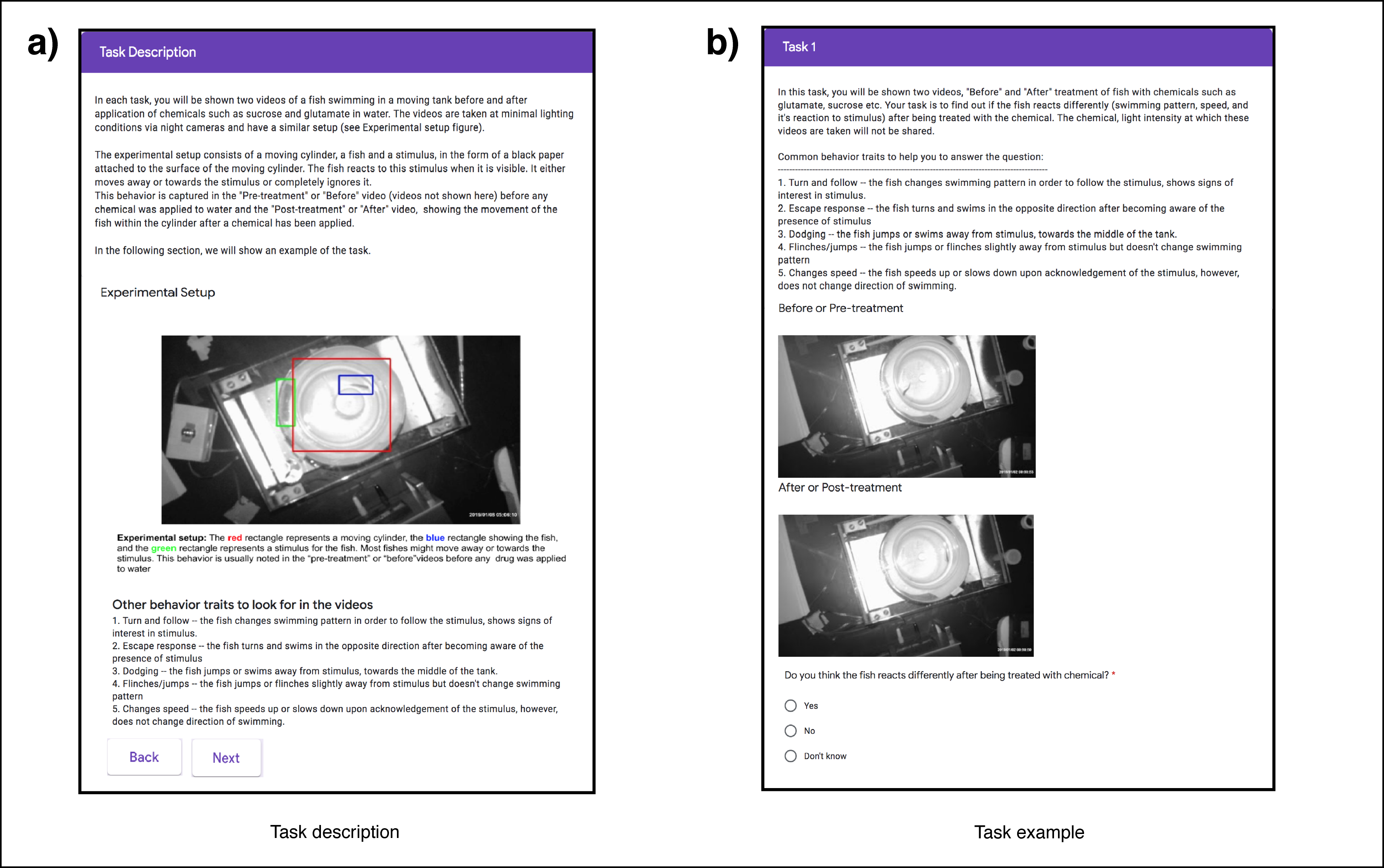

Supplement: Supplementary file 3 — Supplementary Figure 3. [file 41598_2020_79772_MOESM3_ESM.png]
